# First successful implementation of subintimal transcatheter withdrawal technique in intravascular ultrasound–guided tip detection antegrade dissection and reentry: a case report

**DOI:** 10.1093/ehjcr/ytad580

**Published:** 2023-11-20

**Authors:** Shunsuke Kitani, Etsuo Tsuchikane, Masaru Yamaki, Yasumi Igarashi

**Affiliations:** Department of Cardiology, Sapporo Kosei General Hospital, Kita 3-jo Higashi 8-chome, Chuo-ku, Sapporo, Hokkaido 0600033, Japan; Department of Cardiology, Toyohashi Heart Center, Aichi, Japan; Department of Cardiology, Sapporo Kosei General Hospital, Kita 3-jo Higashi 8-chome, Chuo-ku, Sapporo, Hokkaido 0600033, Japan; Department of Cardiology, Sapporo Kosei General Hospital, Kita 3-jo Higashi 8-chome, Chuo-ku, Sapporo, Hokkaido 0600033, Japan

**Keywords:** Percutaneous coronary intervention, Chronic total occlusion, Tip detection antegrade dissection and reentry, Intravascular ultrasound, Subintimal transcatheter withdrawal technique, Case report

## Abstract

**Background:**

Antegrade dissection and reentry (ADR) is an effective technique for wire passage in chronic total occlusion (CTO), and in recent years, the effectiveness of intravascular ultrasound (IVUS)–guided tip detection (TD)-ADR has been reported. However, the expansion of the subintimal space serves as a significant obstacle to the success of ADR, posing a limitation to the procedure.

**Case summary:**

We present the first case of using IVUS-guided TD-ADR with the subintimal transcatheter withdrawal (STRAW) technique. The patient was a 68-year-old Asian female with effort angina pectoris and a CTO in the middle section of the right coronary artery (RCA). Two previous attempts at percutaneous coronary intervention (PCI) for the RCA at another hospital were unsuccessful. During the third attempt PCI, the antegrade wire migrated into the subintimal space. To address this, we performed IVUS-guided TD-ADR using the Conquest Pro 12 Sharpened Tip (CP12ST; Asahi Intecc, Aichi, Japan) wire. However, due to the expansion of the subintimal space, we were unable to puncture the true lumen. To reduce the subintimal space, we employed the STRAW technique, which allowed successful puncture of the true lumen using the CP12ST wire. Finally, stenting was performed, resulting in satisfactory antegrade blood flow.

**Discussion:**

Intravascular ultrasound–guided TD provides accurate guidance for puncturing in ADR procedures, but the expansion of the subintimal space remains a significant challenge. The STRAW technique offers a solution by reducing the subintimal space and enabling successful puncture of the true lumen during IVUS-guided TD-ADR.

**Learning points**

- Intravascular ultrasound–guided tip detection and antegrade dissection and reentry is a reliable technique that has emerged recently for chronic total occlusion, but enlargement of the subintimal space is still a major limitation.

## Introduction

Over the past decade, the success rate of percutaneous coronary intervention (PCI) for chronic total occlusion (CTO) has improved,^1^ although failures still occur, particularly in complex cases.^2^ As a method of guidewire passage for the CTO lesion, antegrade dissection and reentry (ADR) techniques have been reported with several manners, and Stingray balloon (Boston Scientific, Natick, MA) is most commonly employed.^3,4^ Intravascular ultrasound (IVUS)–guided tip detection (TD)-ADR has been reported as an effective method for CTO PCI.^5^ However, the expanded subintimal space hinders successful ADR penetration. This issue is still one of the limitations of ADR procedure. Some case reports highlighted the utility of the subintimal transcatheter withdrawal (STRAW) technique during ADR procedures using a Stingray balloon.^6^ Here, we present the first reported case of successful IVUS-guided TD-ADR with intraoperative expansion of the subintimal space, overcome by the utilization of the STRAW technique.

## Summary figure

**Table:** 

Time period	Event
18 months ago	Onset of chest pain on exertion.
10 months ago	Diagnosed with effort angina pectoris based on symptoms, further evaluation through cardiac computed tomography revealed severe coronary artery disease.
8 months ago	Coronary angiography revealed 75% stenosis in the proximal left anterior descending artery (LAD) and CTO in the middle portion of the right coronary artery (RCA). Left anterior descending artery was treated with a drug-eluting stent, but antegrade PCI for RCA failed at another hospital.
2 months ago	Second attempt at RCA CTO PCI also failed.
Day 1	Admission to our hospital.
Day 2	Scintigraphy showed ischaemia and viability in the inferior wall.
Day 3	Successful third attempt for RCA CTO PCI.
Day 4	No significant increase in myocardial enzyme.
Day 6	Discharged in a favourable clinical course.
1month later	Follow-up revealed complete relief of symptoms.

## Case presentation

**Figure 1:**
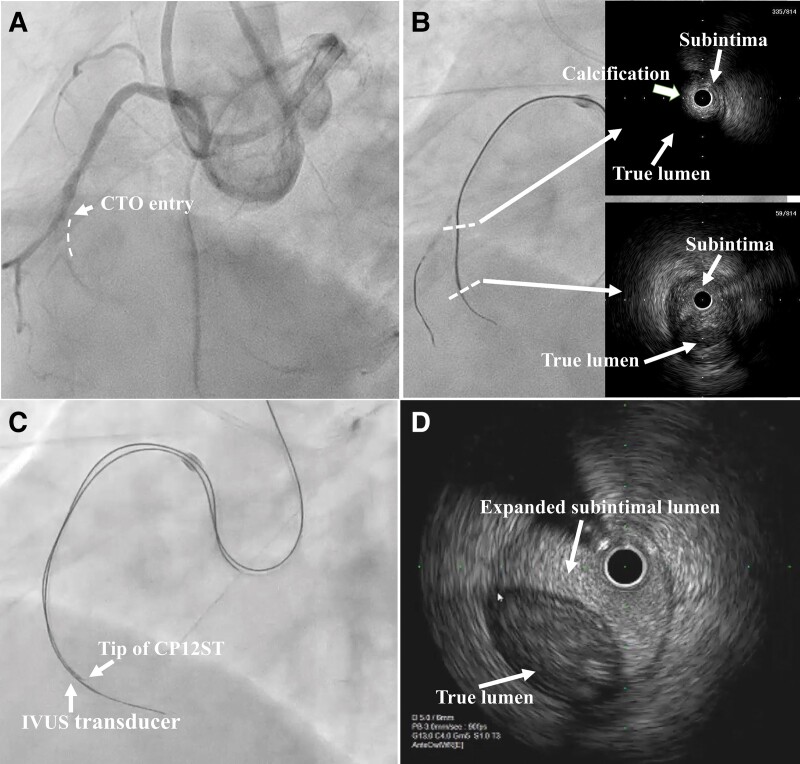
Simultaneous coronary angiogram and intravascular ultrasound assessment in the chronic total occlusion. (*A*) Coronary angiogram. (*B*) Intravascular ultrasound examination from subintimal space. (*C*) Intravascular ultrasound–guided tip detection. (*D*) Intravascular ultrasound image showing subintimal space expansion. CTO, chronic total occlusion; IVUS, intravascular ultrasound; CP12ST, Conquest Pro 12 Sharpened Tip.

A 68-year-old Asian female with a medical history of hypertension, diabetes mellitus, and dyslipidaemia presented with effort angina pectoris caused by a CTO in the middle portion of the RCA (*Figure 1A*). She had undergone successful PCI for the LAD, but the RCA CTO had been unsuccessfully treated in two prior attempts at another hospital. As a result, she was admitted to our hospital for retreatment of the RCA CTO. The patient was mobile and receiving antianginal therapy, including bisoprolol fumarate, amlodipine, nicorandil, and isosorbide dinitrate, along with dual antiplatelet therapy. Physical examination revealed blood pressure of 121/51 mmHg and a heart rate of 69 b.p.m. No cardiac murmurs or pulmonary rales were detected. The electrocardiogram showed no significant changes. Echocardiography indicated a 65% ejection fraction and normal left ventricular contraction, with no significant valvular disease. Blood tests showed a creatinine level of 0.63 mg/dL (normal <0.70 mg/dL) and a brain natriuretic peptide level of 34.0 pg/dL (normal <18.4 pg/dL). Technetium-99m tetrofosmin scintigraphy demonstrated reduced uptake in the inferior wall during pharmacological stress, while uptake at rest was normal. Effort angina pectoris due to the CTO lesion was diagnosed based on echocardiography and scintigraphy, which confirmed the viability of the inferior wall. The J-CTO score for this patient was 3 points, attributed to factors including calcification, blunt occlusion, and being a retry case.^7^

Due to the anatomical complexity and it being the third attempt, we initially started with a retrograde approach. However, it was evident that the septal channels were the primary conduits for collateral circulation, which unfortunately did not appear promising due to their misty appearance. We diligently attempted to access several of these misty septal channels, hoping to establish a retrograde strategy. Unfortunately, at the distal segments of these channels, we encountered significant tight bends, and we were regrettably unable to achieve successful passage through any of them. Consequently, we opted to transition to an antegrade approach for this RCA CTO PCI procedure. An 8-French AL1.5SH catheter was engaged in the RCA through the femoral artery. AnteOwl WR (Terumo, Tokyo, Japan) IVUS (AO-IVUS) examination revealed a heavily calcified cap at the CTO entry. We attempted to advance the Ultimate Bros 3 (Asahi Intecc, Aichi, Japan) wire with the Corsair Pro microcatheter (Asahi Intecc, Aichi, Japan) into the CTO lesion, but it appeared to have migrated into the subintimal lumen. Despite attempting the balloon anchor technique, we were unable to advance the AO-IVUS catheter into the CTO lesion. After expanding the CTO lesion using a 1.5 mm ZINRAI (Kaneka Corp., Osaka, Japan) balloon, we introduced the AO-IVUS catheter, confirming its positioning within the subintimal space. The IVUS examination, which was performed in the subintimal space, provided information that the penetration difficulty from the subintima to the true lumen emerged due to extensive calcification. However, in a part of the vessel, which had relatively lower calcification, there appeared to be a potential for penetration from the subintima to the true lumen (*Figure 1B*).

Subsequently, we performed TD-ADR using the Conquest Pro 12 Sharpened Tip (CP12ST) wire (Asahi Intecc, Aichi, Japan) in combination with the FinecrossGT microcatheter (Terumo, Tokyo, Japan; *Figure 1C*). Despite our efforts, penetration into the distal true lumen was unsuccessful due to the expanded subintimal space (*Figure 1D*). Recognizing the difficulty of penetration with an enlarged subintimal space, we decided to perform the STRAW technique.

**Figure 2:**
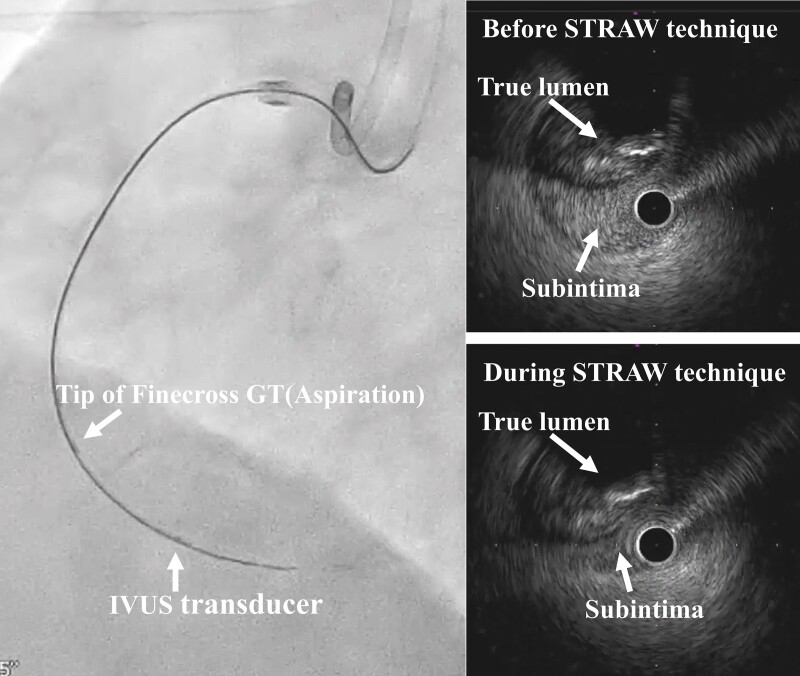
Intravascular ultrasound images before and during the subintimal transcatheter withdrawal technique. IVUS, intravascular ultrasound; STRAW, subintimal transcatheter withdrawal.

**Figure 3:**
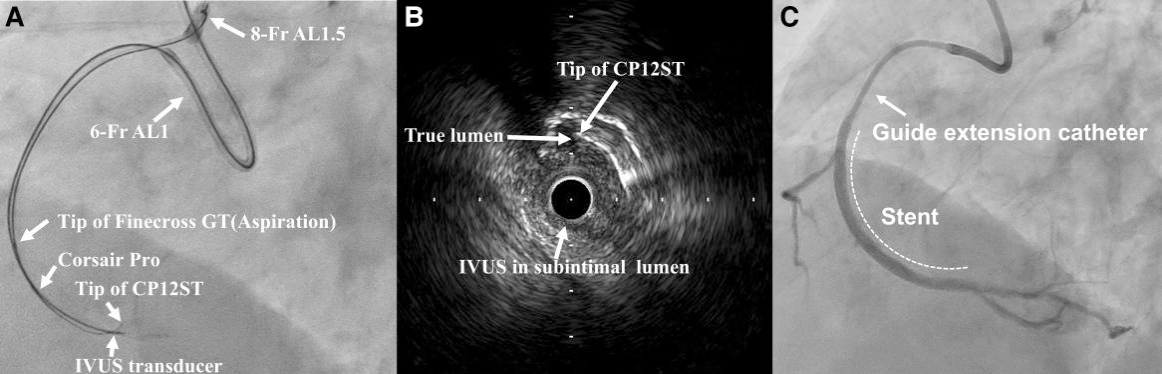
Successful reentry using intravascular ultrasound–guided tip detection under the subintimal transcatheter withdrawal technique. (*A*) Intravascular ultrasound–guided tip detection antegrade dissection and reentry under the subintimal transcatheter withdrawal technique. (*B*) Intravascular ultrasound image of successful penetration from the subintimal lumen to the true lumen using Conquest Pro 12 Sharpened Tip wire. (*C*) Final angiogram. CP12ST, Conquest Pro 12 Sharpened Tip; IVUS, intravascular ultrasound.

The subintimal aspiration using the FinecrossGT microcatheter was performed to decompress the enlarged subintimal space, which was confirmed with IVUS (*Figure 2*, Supplementary material online, *Video S1*). To facilitate IVUS-guided TD-ADR while continuing aspiration, a double-guiding catheter system was constructed using a 6-French AL1SH catheter engaged with the RCA. Using this 6-French guiding catheter, the Corsair Pro microcatheter was advanced to the subintimal space. Subsequently, TD-ADR was performed using the CP12ST wire with the Corsair Pro microcatheter while continuing the STRAW technique and IVUS examination through the 8-French guiding catheter (*Figure 3A*). We successfully penetrated the tip of the CP12ST wire toward the true lumen under IVUS observation (*Figure 3B*, Supplementary material online, *Video S2*). The Corsair Pro microcatheter followed CP12ST into the distal true lumen. We then switched from the CP12ST wire to the Sion wire (Asahi Intecc, Aichi, Japan) and successfully passed through the distal RCA. After dilation with a 2.5 mm balloon, two drug-eluting stents were deployed, resulting in the successful revascularization of the RCA CTO (*Figure 3C*). The procedure took 336 min, with a radiation dose of 4.7 Gy. The prolonged duration was primarily due to the initial pursuit of a time-consuming retrograde approach and challenges in wire control caused by subintimal space expansion during the IVUS-guided TD-ADR procedure. However, the introduction of the STRAW technique under IVUS-guided TD-ADR facilitated successful wire passage in ∼25 min.

The patient was discharged uneventfully on postoperative Day 3. Upon discharge, nicorandil was discontinued. Additionally, during the 1-month follow-up after PCI, isosorbide dinitrate was also phased out. These decisions were driven by the complete resolution of the patient’s symptoms. This adjustment in medication regimen serves as a positive indicator of the intervention’s efficacy.

## Discussion

**Figure 4:**
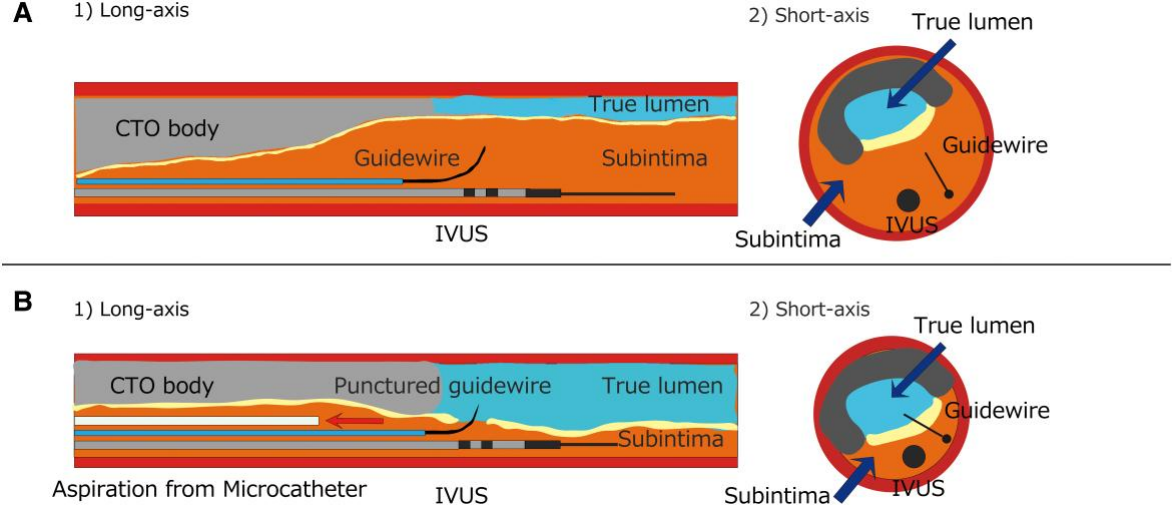
Illustration of tip detection antegrade dissection and reentry under the subintimal transcatheter withdrawal technique. (*A*) Before the subintimal transcatheter withdrawal technique. The guidewire cannot be controlled under the expanded subintimal space. (*B*) During the subintimal transcatheter withdrawal technique. Aspiration through a microcatheter reduces the subintimal space, making penetration from subintima to true lumen easier with a stiff guidewire. CTO, chronic total occlusion; IVUS, intravascular ultrasound.

Several reentry techniques have already been reported, and Stingray-ADR is the standard technique used worldwide for reentry procedures in CTO PCI.^1,3,4^ Intravascular ultrasound–guided TD-ADR has shown efficacy in CTO PCI by providing real-time visualization of the target lumen and guidewire tip, enabling precise puncture of the distal true lumen.^5^ Recent case reports have demonstrated the effectiveness of CP12ST in Stingray-ADR^8^ and TD-ADR procedures.^8,9^ However, once subintimal space expansion occurs, those ADR procedures will encounter difficulties with wire puncture, even with high penetration force wires like CP12ST (*Figure 4A*).

The STRAW technique offers a viable option for decompressing the distal true lumen and enabling successful reentry in CTO cases.^6,10^ In our case, we utilized the STRAW technique, involving the placement of a microcatheter within the subintimal space and continuous aspiration, to effectively manage the expanding subintimal space. This approach not only enabled real-time monitoring of the reduction in subintimal space using IVUS during TD-ADR but also improved the manoeuvrability and control of the guidewire, facilitating more precise puncture into the distal target true lumen (*Figure 4B*).

This approach has several limitations. A double-guiding catheter system is necessary to perform this procedure due to the inability to insert the AO-IVUS and two microcatheters even through an 8-French guiding catheter. It is important to note that this procedure becomes difficult when there is a lesion at the ostium of the coronary artery. One potential limitation is that the current approach does not effectively inhibit the in-flow, which could result in an insufficient reduction of the subintimal space with microcatheter aspiration alone. In such a scenario, instead of using a microcatheter, employing an over-the-wire balloon to expand and control inflow while simultaneously performing aspiration through the wire lumen of the balloon may offer a potential solution to reduce subintimal expansion.

## Conclusion

Intravascular ultrasound–guided TD-ADR is a reliable technique for CTO PCI; however, the expansion of the subintimal space still presents a limitation. The addition of the STRAW technique to IVUS-guided TD-ADR suggests a potential benefit in facilitating guidewire penetration into the distal true lumen when subintimal expansion occurs. Further research and experience are needed to investigate the effectiveness of this combined approach in a general CTO lesion.

## Supplementary Material

ytad580_Supplementary_Data

## Data Availability

The data underlying this article are available in the article and in its online Supplementary material.

## Lead author biography

Dr Shunsuke Kitani was born in Kanagawa, Japan, in 1979 and graduated from Hokkaido University in 2005. He trained in cardiology at Sapporo City General Hospital, JCHO Hokkaido Hospital, and Tokeidai Memorial Hospital. He is currently working at Sapporo Kosei General Hospital as the Director of Cardiology. He specializes in complex cardiovascular interventions.
